# Canted spin order as a platform for ultrafast conversion of magnons

**DOI:** 10.1038/s41586-024-07448-3

**Published:** 2024-05-29

**Authors:** R. A. Leenders, D. Afanasiev, A. V. Kimel, R. V. Mikhaylovskiy

**Affiliations:** 1 Department of Physics, Lancaster University, Lancaster, UK; 2 Radboud University, Institute for Molecules and Materials, Nijmegen, The Netherlands

**Keywords:** Magnetic properties and materials, Magneto-optics, Spintronics

## Abstract

Traditionally, magnetic solids are divided into two main classes—ferromagnets and antiferromagnets with parallel and antiparallel spin orders, respectively. Although normally the antiferromagnets have zero magnetization, in some of them an additional antisymmetric spin–spin interaction arises owing to a strong spin–orbit coupling and results in canting of the spins, thereby producing net magnetization. The canted antiferromagnets combine antiferromagnetic order with phenomena typical of ferromagnets and hold great potential for spintronics and magnonics^1–5^. In this way, they can be identified as closely related to the recently proposed new class of magnetic materials called altermagnets^6–9^. Altermagnets are predicted to have strong magneto-optical effects, terahertz-frequency spin dynamics and degeneracy lifting for chiral spin waves^10^ (that is, all of the effects present in the canted antiferromagnets^11,12^). Here, by utilizing these unique phenomena, we demonstrate a new functionality of canted spin order for magnonics and show that it facilitates mechanisms converting a magnon at the centre of the Brillouin zone into propagating magnons using nonlinear magnon–magnon interactions activated by an ultrafast laser pulse. Our experimental findings supported by theoretical analysis show that the mechanism is enabled by the spin canting.

## Abstract (web summary)

A study demonstrates a new functionality of canted spin order for magnonics and shows that it facilitates mechanisms for ultrafast nonlinear conversion of magnons.

## Main

Very recently, the existence of a new distinct magnetic material class, altermagnets, was proposed in addition to the conventional ferromagnets and antiferromagnets^10^. Altermagnets have antiparallel spin alignment with either zero or vanishing magnetization, but behave as if they have a large magnetic moment by showing, for instance, giant Faraday or Hall effects. Therefore, altermagnets are predicted to be very similar to materials that were traditionally known as antiferromagnets with spin canting or weak ferromagnets.

Among the latter, the orthoferrites evince a coupling between the ferromagnetic moment **M** and the antiferromagnetic Néel vector **L**, described by the free-energy term

1 $${\varPhi }_{{\rm{DMI}}}={\bf{D}}\cdot \left({\bf{M}}\times {\bf{L}}\right),$$

in which the vector **D** characterizes the Dzyaloshinskii–Moriya antisymmetric exchange interaction. In the paradigm-shifting papers that introduce altermagnetism, the orthoferrites have been identified as examples of altermagnets^5,10^ with potentially strong nonlinearities of their spin dynamics. Thus, orthoferrites combine the altermagnetic symmetry with canted spin ordering, leading to the giant magneto-optical responses characteristic of the altermagnets^13^, together with high resonance frequencies. This unique combination makes them promising candidates for the field of magnonics, which is concerned with the investigation of (propagating) spin waves, or magnons.

Magnonics aims to use quanta of spin waves, magnons, to carry, transport and process information, avoiding the dissipation of energy inherent to electronics^14,15^. However, despite the successful demonstration of magnon-based logic operations^16^, a magnon transistor^17^ and the achievement of magnon frequency multiplication^18^, so far the performance of magnonics has been restricted to the gigahertz frequency range and millimetre-size devices. The use of antiferromagnetic materials instead of conventional ferromagnets represents a breakthrough because of the very high (terahertz) frequency, nanometre-scale wavelength and fast velocities of their magnons^1,2,19^. Recent experiments on electric control and detection of antiferromagnetic oxides have hinted at tantalizing opportunities in this area^20–22^. However, progress in high-frequency magnonics, and development of magnonic logic in particular, has been held back by the lack of coherent sources of propagating terahertz spin waves. As a consequence, investigation of magnon propagation in antiferromagnets is still in its infancy^3,4,23–25^. Recently it was demonstrated that ultrashort pulses of light can be a game-changer in this quest. It has been shown that in the iron oxides the above-bandgap ultrashort photoexcitation acts akin to a nanoscale spin-wave source emitting an ultrabroadband terahertz packet of coherent magnons^26^.

## Concept of up-conversion of magnons

Here we show that the coupling between **M** and **L** present in orthoferrites enables a new mechanism for nonlinear interaction between magnons, activated by an external excitation. Normally, the torque **T** acting on a spin system is given by the cross product of the magnetization and the effective magnetic field **H**_eff_, such that ${\bf{T}}\propto {\bf{M}}\times {{\bf{H}}}_{{\rm{e}}{\rm{f}}{\rm{f}}}$. However, if the spins are out of equilibrium, a coupling between the spin deflections and the effective field arises, which acts as an additional nonlinear torque with its strength $\propto \varphi D{H}_{{\rm{e}}{\rm{f}}{\rm{f}}}.$ This torque is proportional to the effective field, the strength of the Dzyaloshinskii–Moriya interaction *D*, and the spin deflection *φ* from the equilibrium orientation (Methods). One may exploit this torque to realize the nonlinear regime of magnon propagation and convert a magnon into another, with a different momentum and energy. To demonstrate the feasibility and efficiency of this mechanism, we achieve the nonlinear conversion of an optically triggered magnon at the centre of the Brillouin zone into propagating coherent magnons in an archetypical canted antiferromagnet with altermagnetic-symmetry holmium orthoferrite, HoFeO_3_ (see Methods for more details).

**Fig. 1:**
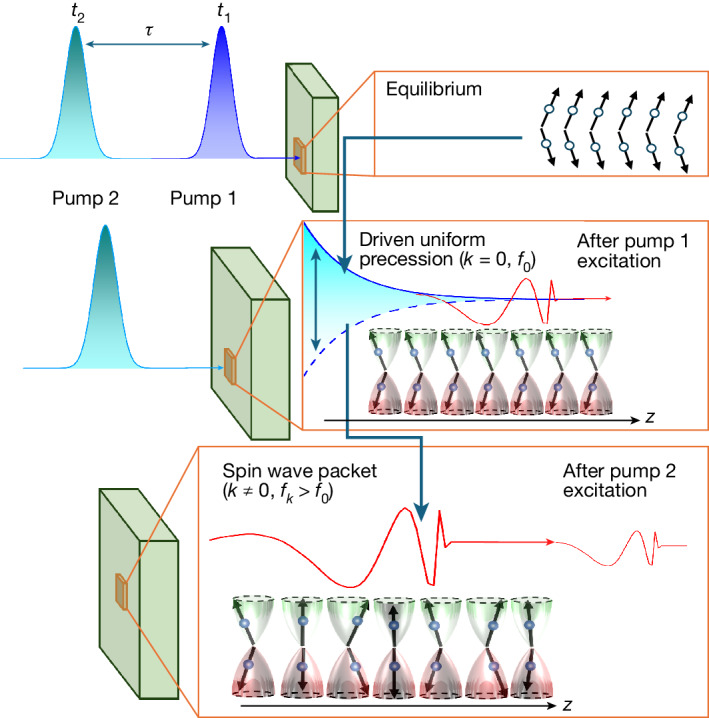
Schematic of the up-conversion of the quasi-uniform precession to the propagating magnon modes. Top: before the arrival of the first pump, the spin system is in equilibrium. Middle: the first pump, arriving at *t* = *t*_1_, excites spin dynamics, consisting of the quasi-uniform precession with intrinsic resonance frequency *f*_0_ (blue) and a propagating magnon wave packet with higher frequencies (red). Bottom: at the time of arrival of the second pump *t*_2_ = *t*_1_ + *τ*, the wave packet excited by pump 1 has propagated away, and the remaining quasi-uniform precession is converted into an amplified spin wave through the nonlinear torque, which is proportional to the spin deflection induced by the first pump.

To reveal this process, we use the new double-excitation technique illustrated in Fig. 1. The first pump pulse has a photon energy above the bandgap energy *E*_g_ of HoFeO_3_ of about 3 eV. Consequently, the photons are strongly absorbed and the optical excitation is localized near the surface of the material. This confined excitation generates a broadband wave packet of the coherent spin waves, which propagate normal to the boundary^26^. At the same time, the pump drives the strong quasi-uniform precession in the region of the laser field confinement, which in contrast does not propagate. It corresponds to a forced response by the driving effective field of the laser excitation^27^. Here we show that this non-stationary and strongly non-uniform magnetization response can be up-converted into propagating magnons with higher frequencies (energies) and wave numbers (momenta) using a second optical pump pulse.

## Propagating magnon modes in HoFeO_3_

**Fig. 2:**
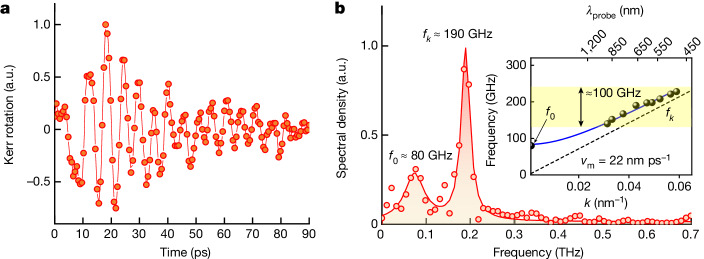
Observation of the magnon modes in the single-pump experiment. **a**, Single-pump–probe MOKE signal for a probe wavelength of 660 nm. The signal is normalized and the non-oscillatory background signal is subtracted. **b**, Fourier transform of the data in **a**. The spectrum is fitted with the sum of two Lorentzian functions. Inset: the retrieved magnon dispersion, which is fitted with the solid blue line based on equation (1). a.u., arbitrary units. Source Data

**Extended Data Fig. 1:**
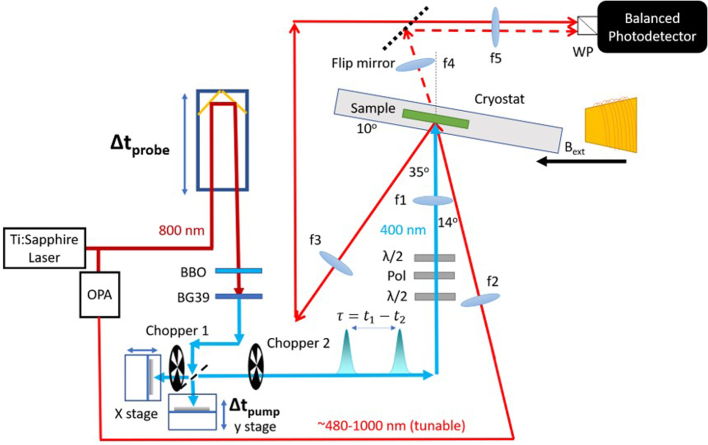
Schematic of the experimental setup. More details on the setup are given in the section “Experimental Setup” in the Methods.

First we carried out the optical pump–probe experiment in which we excite a broadband magnon wave packet propagating along the normal of the antiferromagnet interface using 400-nm light $({hv} > {E}_{{\rm{g}}})$^28^ (see Methods and Extended Data Fig. 1 for a schematic of the setup). To selectively detect individual spectral components of the wave packet, we tracked time-resolved dynamics of the magneto-optical Kerr effect (MOKE) varying the wavelength of the probe pulse in the visible spectral range. Figure 2a shows the pump-induced high-frequency magnetic dynamics measured with a probe pulse at a wavelength *λ*_pr_ = 660 nm. We analysed this signal in the Fourier domain (Fig. 2b) and found that the signal has two clear features around 190 GHz and 80 GHz. We attributed the signal at *f*_*k*_ = 190 GHz to the finite-*k* magnon mode, selectively detected from the propagating spin wave packet, and the oscillation at *f*_0_ = 80 GHz to the *k* = 0 quasi-uniform precession mode. These frequencies are related through the antiferromagnetic dispersion relation^29^:

2 $${\omega }_{k}^{2}={\omega }_{0}^{2}+{v}_{{\rm{m}}}^{2}\,{k}_{{\rm{m}}}^{2},$$

in which ${\omega }_{k}=2{\rm{\pi }}{f}_{k}$ is the angular frequency of the magnon with wave number *k*_m_, ${\omega }_{0}=2{\rm{\pi }}{f}_{0}$ is the angular frequency of the quasi-uniform precession mode, and *v*_m_ is the relativistic velocity limit for the propagating magnons in an antiferromagnet. The detected magnon wave number *k* is related to the normal projection of the probe wave vector through the Brillouin condition:

3 $${k}_{{\rm{m}}}=2{k}_{{\rm{pr}}}=\frac{4{\rm{\pi }}n\,\cos \left(\beta \right)}{{\lambda }_{{\rm{pr}}}}=0.04\,{{\rm{nm}}}^{-1}.$$

Here $n\approx 2.23$ is the refractive index and $\beta \approx 2{5}^{^\circ }$ is the angle of incidence of the probe pulse with a wavelength of *λ*_pr_. This wave number corresponds to a spin wave having a wavelength of approximately 150 nm. By varying the probe wavelength and tracking the detected magnon frequency, we mapped out the spin-wave dispersion in HoFeO_3_ (inset Fig. 2b). We found that it agrees well with the one reported in ref. ^30^ and provides *v*_m_ = 22 nm ps^−1^. We estimate the typical pump-induced spin deflections in our experiments to be large, reaching up to approximately 15° (Extended Data Fig. 3).

Additionally, we investigated the excitation mechanism of the propagating spin waves by varying the linear polarization of the incident pump. Our observed polarization dependence closely resembles the one expected for the inverse Cotton–Mouton effect^31^. Thus, one may conclude that the spin waves are non-thermally excited (Extended Data Fig. 4). Moreover, we note that in the altermagnets the magnon dispersion branches are split for different chiralities^32^, which in the simplest case are circularly polarized modes. In the orthoferrites, the complex magnetic anisotropy results in the splitting of the magnon modes in the quasi-ferromagnetic and quasi-antiferromagnetic branch, corresponding to the elliptical precession of the ferromagnetic vector and antiferromagnetic vector, respectively. These magnons are known to carry angular momentum and are therefore chiral^24^. In our experiment, we observed the magnons lying only on the quasi-antiferromagnetic branch.

## Evidence for up-conversion of magnons

**Fig. 3:**
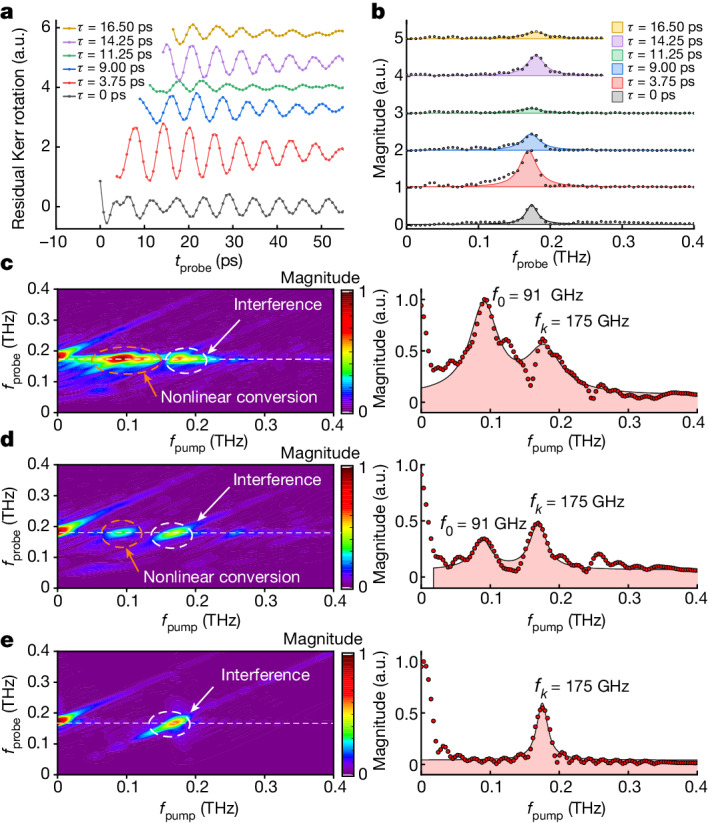
Demonstration of the nonlinear conversion in the 2D spectroscopy experiment. **a**, Normalized probe polarization rotation traces, for various delays between the two pump pulses. Both of the pumps are modulated, and the data are shown after the arrival of the second pump pulse. The non-oscillatory background signal is subtracted. **b**, Fourier transforms of the traces in **a**. **c**, Left: 2D Fourier transform of the data measured in **a**, with the Fourier transform along the probe delay *t* on the vertical axis and the Fourier transform along the pump delay *τ* on the horizontal axis. Right: the spectrum along the pump frequency at the magnon frequency detected by the probe (indicated by the white dashed line in the left panel). **d**, Similar spectrum to **c**, but with the Fourier transform carried out on the data only after *τ* = 14 ps pump delay. **e**, Left: reference 2D Fourier transform obtained by adding two single-pump reference scans. Right: plot of the cross-section at the dashed white line in the left panel. Source Data

After the characterization of the propagating magnon modes in HoFeO_3_, we introduced the second pump pulse by splitting the 400-nm pump in half in a variable-length Michelson interferometer arm to excite the sample at a variable time delay $\tau ={t}_{1}-{t}_{2}$ with respect to the first pump pulse^33^ (Methods and Extended Data Fig. 1). Again, we carried out the pump–probe measurements with a 660-nm probe pulse, and investigated how the dynamics was affected at the various *τ*. For the initial double-pump–probe experiment, we designed a configuration to demonstrate coherent control of the spin-wave amplitude. We did this by sending both pump pulses through the same optical modulator (chopper), such that both pumps induce a signal with the same modulation frequency. As a result, the sum of the two pump-induced signals should be detected. From the measured signals shown in Fig. 3a,b, one can see that the signals not only are either suppressed or amplified but also change their phase or sign, depending on the time delay between the two pulses (see also Extended Data Fig. 5 for examples of comparisons of the dynamics before and after arrival of the second pump), indicating that the response cannot be ascribed to interference due to simple linear superposition. Indeed, analysis of the two-dimensional (2D) spectrum (Fig. 3c) demonstrates further that the amplitude of the oscillation at the magnon frequency *f*_*k*_ is modulated beyond coherent control through interference. The 2D frequency map features two maxima observed at diagonal frequencies $({f}_{k}\,,{f}_{k}\,)$, and off-diagonal frequencies $({f}_{0}\,,{f}_{k}\,)$. We note that the *k* = 0 mode at frequency *f*_0_ in the spectrum is broad, and thus strongly damped. We checked this damping by processing the data after the typical lifetime of the *k* = 0 mode (*τ* > 14 ps). Indeed, we see that the $({f}_{0}\,,{f}_{k}\,)$ peak is suppressed for longer delay times between the two pumps (Fig. 3d). One can attribute the diagonal feature to the interference due to so-called coherent control^34^, as is confirmed by adding two separate single-pump responses for variable time delays between these two pumps (see Fig. 3e and Methods for more details). However, the linear superposition of the two responses cannot explain the presence of the off-diagonal feature, which we interpret as a signature of nonlinearity, corresponding to the up-conversion of the quasi-uniform precession mode *f*_0_ to the propagating magnon mode *f*_*k*_. It is important to note that the $({f}_{0}\,,{f}_{k}\,)$ peak is stronger than the $({f}_{k}\,,{f}_{k}\,)$ one, testifying to the strongly nonlinear regime of magnon propagation.

To estimate the amplitude of the optical conversion from the *k* = 0 magnons to *k* ≠ 0 magnons, compared to that of the direct excitation of the *k* ≠ 0 magnons, we process the data presented in Fig. 3 using a 1D Fourier transform for the pump delay. In Extended Data Fig. 6, the magnitude and real and imaginary parts of the Fourier transform at frequency *f*_*k*_ are shown as a function of the pump delay. From this, we estimate that the amplitude of the conversion is of the same order of magnitude as that of the direct excitation of propagating magnons.

To confirm our interpretation, we repeated the experiment with similar conditions; however, now modulating only one of the pumps with the optical chopper, such that only the effect of the second pump on the dynamics excited by the first pump becomes measurable and the linear interference becomes undetectable. We observed the same features in the 2D map as in Fig. 3 (see also Extended Data Fig. 7). The fact that the diagonal peak remains implies that nonlinear modulation of the propagating magnon mode occurs in addition to the modulation by the linear superposition of the two excitations.

In addition, similar measurements were carried out for different conditions, by varying the pump fluences and probe wavelength, as shown in Extended Data Fig. 8. We consistently observe that the peaks in the 2D spectra appear at the diagonal $({f}_{k}\,,{f}_{k}\,)$ frequencies and the off-diagonal $({f}_{0}\,,{f}_{k}\,)$ frequencies. Moreover, we have repeated the measurements at different temperatures and noted that the nonlinearity is most pronounced at the temperature of *T* = 44 K, at which ${f}_{k}\approx {2f}_{0}$ and hence the nonlinear conversion is enhanced. This temperature also corresponds to the maximal amplitudes in the single-pump experiment.

## Theoretical model and simulations

To describe the observed nonlinear behaviour, we have developed a model by deriving the Klein–Gordon equation for spin dynamics in the orthoferrites, and retaining not only the linear terms^27^, but also the terms that involve a coupling between the spin deflection and the light-induced effective field *h*, responsible for the excitation of the magnons. We find that, owing to this coupling, the second pump pulse exerts an additional nonlinear torque on the spin system, which is proportional to the Dzyaloshinskii–Moriya interaction parameter *D* and the non-equilibrium spin deflection, which is induced by the first pump pulse (Methods). In our model, we assume that the pump pulses are confined in a thin region of thickness *d*. The localization of the second pump pulse in the region of the thickness *d* is essential for the generation of the broadband packet of magnons and for the up-conversion of the quasi-uniform precession excited by the first pump pulse, which persists only in this region. The range of *k* vectors that can be generated depends on the excitation thickness *d*, $\Delta k\approx \frac{2{\rm{\pi }}}{d}$, and is largest if the second pump pulse is also strongly confined. As the canting angle in orthoferrites is proportional to *D*, this nonlinearity is expected to be most pronounced in the antiferromagnets and altermagnets with large canting angles and absent in ferromagnets. Moreover, the Dzyaloshinskii–Moriya interaction can have a different strength at the sample interface and thus induce additional spin canting, which may contribute to the nonlinear conversion as well.

**Fig. 4:**
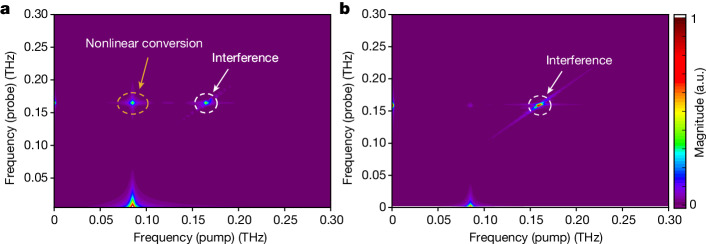
Numerical simulations of the double-pump MOKE experiment. **a**,**b**, Calculated 2D spectrum for experimentally relevant parameters, for large spin deflections *φ* of 10° (**a**) and small deflections of 1° (**b**). Source Data

We use our model for the magneto-optical detection of propagating magnons^27^ including this nonlinear torque for varying arrival times of the second pump. The simulation of the 2D MOKE spectrum is depicted in Fig. 4a.

In the simulations, we observe the emergence of the two peaks at the diagonal frequency and the off-diagonal frequency. We assume the quasi-uniform precession frequency of *f*_0_ = 100 GHz and calculate that a probe pulse at a wavelength of 660 nm is predicted to detect the magnon mode with *f*_*k*_ *=* 170 GHz. As in the experiment, we see two peaks, one at the diagonal position $({f}_{k}\,,{f}_{k}\,)$ and another at the off-diagonal position $({f}_{0}\,,{f}_{k}\,)$. The frequency chosen in the calculation *f*_0_ = 100 GHz is slightly redshifted to the experimentally observed frequency of approximately 85 GHz owing to the inhomogeneity of the spin–spin interaction after excitation with the light pulse (Methods). As a result, we find that for experimentally relevant parameters, the proposed model matches the experimental results very well. Moreover, we have carried out the simulations for the case when the spin deflections are an order of magnitude smaller than the experimentally observed deflections, and see that this nonlinear conversion feature disappears, as shown in Fig. 4b. Thus, this nonlinear conversion can occur only for sufficiently high spin deflections induced by the first pump pulse. As our model shows that the magnitude of the nonlinearity scales linearly with the Dzyaloshinskii–Moriya interaction, we expect this effect to be most pronounced in spin systems with large canting angles, thus making the broad class of altermagnets with canted spins (orthoferrites, haematite, iron borate and so on) a suitable class of materials for the investigation of this effect. Finally, we consider other potential mechanisms for nonlinearity that, for instance, directly arise from large spin deflections *φ*^2^, *φ*^3^ and so on. Indeed, such terms have been reported to be responsible for the generation of higher harmonics and a cross-talk between the quasi-ferromagnetic and the quasi-antiferromagnetic modes in the orthoferrites^35,36^. However, as neither of these effects is seen in our data, we can rule out these mechanisms of nonlinearity as being responsible for the observed up-conversion.

## Conclusion and outlook

In conclusion, we have demonstrated a strongly nonlinear effect in the excitation of propagating magnons in HoFeO_3_, by carrying out double-pump–probe ultrafast spectroscopy experiments. We found that the additional torque induced by the coupling of a light pulse with already excited antiferromagnetic precession is the origin of this nonlinearity, and is governed by the Dzyaloshinskii–Moriya interaction. We modelled the 2D MOKE experiment including this nonlinear torque, and found an accurate qualitative agreement with the experimental data. Although recent breakthroughs highlighted the importance of interactions between light-induced magnetic, phononic and electronic excitations in the terahertz range^37–41^, most of these experiments were strictly limited to spin excitation in the centre of the Brillouin zone. Our innovative experimental design and the use of the material combining spin canting with altermagnetism allowed us to observe nonlinear interactions and transformations of magnons not only in energy (frequency), but also in momenta (wave vectors). These results unlock the potential of canted spin ordering in altermagnets for magnonic logic devices operating at terahertz clock rates, for which nonlinear control of the propagating spin waves is of essential importance. As the canted antiferromagnets have the same symmetry as altermagnets and exhibit all of the behaviour predicted for the latter, similar nonlinear mechanisms can be anticipated in the altermagnets without strong spin–orbit coupling, but in which the canting is induced by an external magnetic field. Furthermore, we anticipate that the more complex non-collinear spin configurations such as helical or cycloidal orders may support even stronger nonlinear interactions between magnons.

## Methods

### Theoretical description

#### Derivation of the nonlinear torque

We developed a model for the generation of the propagating magnons in the nonlinear conversion regime, and derive a model for the magneto-optical detection of the spin waves. We start by writing the Landau–Lifshitz equations for antiferromagnetic spin dynamics^42^

4 $$\begin{array}{c}\frac{{\rm{d}}{\boldsymbol{M}}}{{\rm{d}}t}=\gamma \left({\bf{M}}\times \frac{\delta W}{\delta {\bf{M}}}\right)+\gamma \left({\bf{L}}\times \frac{\delta W}{\delta {\bf{L}}}\right),\\ \frac{{\rm{d}}{\bf{L}}}{{\rm{d}}t}=\gamma \left({\bf{M}}\times \frac{\delta W}{\delta {\bf{L}}}\right)+\gamma \left({\bf{L}}\times \frac{\delta W}{\delta {\bf{M}}}\right).\end{array}$$

In equation (4), **M** is the ferromagnetic component **M**_1_ + **M**_2_ and **L** is the antiferromagnetic component **M**_1_ − **M**_2_. The terms $\frac{\delta W}{\delta {\bf{M}}}$ and $\frac{\delta W}{\delta {\bf{L}}}$ represent the internal effective fields in the spin system. *W* denotes the free energy of the spin system and for the orthoferrite is given by

5 $$W=\frac{1}{2}\,J{M}^{2}+D[{M}_{x}{L}_{z}-{M}_{z}{L}_{x}]+\frac{1}{2}({K}_{y}-{K}_{x}){L}_{y}^{2}+\frac{1}{2}({K}_{z}-{K}_{x}){L}_{z}^{2}+\frac{1}{4}{K}_{4}{L}^{4}+{q}^{{\prime} }{(\nabla {\bf{M}})}^{2}+q{(\nabla {\boldsymbol{L}})}^{2}-{\bf{M}}\cdot {\bf{h}}(t),$$

in which *J* is the exchange constant, *D* is the Dzyaloshinskii–Moriya constant, *K*_*x,y,z*,4_ are the magnetic anisotropy constants, *q*′ and *q* are the exchange stiffness constants, and **h**(*t*) is the effective field that drives the spin dynamics. Here $J <  < D <  < {K}_{x,y,z} <  < {K}_{4}$. Note that in equation (4) the damping is neglected for simplicity. We add phenomenological damping to our final equations of motion.

We write the equations of motion for each separate component of the **M** and **L** vectors in the Γ_2_ phase $({{\bf{L}}}_{{\bf{0}}}=(\mathrm{0,0},{L}_{0}),{{\bf{M}}}_{{\bf{0}}}=({M}_{0},\mathrm{0,0}))$, and carry out the linearization procedure, writing the spin deflections as the sum of the static and dynamic magnetizations, and assume that the dynamic component is small:

6 $$\begin{array}{c}{\bf{M}}(t)={{\bf{M}}}_{0}+{\bf{m}}(t),\\ {\bf{L}}(t)={{\bf{L}}}_{{\bf{0}}}+{\bf{l}}(t),\\ \left|{\bf{m}}\left(t\right)\right|\ll \left|{{\bf{M}}}_{{\bf{0}}}\right|,\left|{\bf{l}}\left(t\right)\right|\ll \left|{{\bf{L}}}_{{\bf{0}}}\right|.\end{array}$$

Also, we describe the spin deflection in terms of angular coordinates.

7 $$\begin{array}{l}{l}_{y}(t)={L}_{y}(t)-{L}_{y,0}={L}_{0}\sin (\varphi (t))\approx {L}_{0}\varphi (t),\\ {l}_{z}(t)={L}_{z}(t)-{L}_{0}^{z}={L}_{0}\cos (\varphi (t))\approx {L}_{0}(1-\varphi {(t)}^{2}/2)-{L}_{0}\approx 0.\end{array}$$

From the assumption discussed above, we can neglect all terms containing products *φ*^2^. Typically, the product of the spin deflection and effective field that excites the spin deflection is also neglected, as the spin deflection is *φ* *=* 0 before the excitation. This is, however, not the case at the arrival of the second pump in our experiment, so we retain the terms containing $\varphi (t)h(t).$

As a result, we come to the Klein–Gordon equation with an additional nonlinear torque.

8 $$\frac{{{\rm{\partial }}}^{2}{\varphi }_{2}(z,t)}{{\rm{\partial }}{t}^{2}}+({\omega }_{0}^{2}-{v}_{{\rm{s}}{\rm{w}}}^{2}{{\rm{\nabla }}}^{2}){\varphi }_{2}(z,t)+2\alpha \frac{{\rm{\partial }}{\varphi }_{2}(z,t)}{{\rm{\partial }}t}=-{\omega }_{h}\frac{{\rm{\partial }}{h}_{x}(z,t)}{{\rm{\partial }}t}+{\omega }_{D}{\omega }_{h}{\varphi }_{1}(z,t){\mathop{h}\limits^{ \sim }}_{2}(z,t).$$

Here *v*_sw_ is the spin-wave propagation velocity and *α* is the damping parameter. We obtained this simplified expression by introducing the following parameters:

9 $$\begin{array}{c}\,\,{\omega }_{0}=\sqrt{{\omega }_{{\rm{E}}}{\omega }_{{\rm{A}}}},\\ \,\,{\omega }_{{\rm{E}}}=\gamma {L}_{0}\,J,\\ \,\,{\omega }_{{\rm{A}}}=\gamma {L}_{0}({K}_{{\rm{z}}}-{K}_{{\rm{x}}}),\\ \,\,{\omega }_{D}=\gamma {L}_{0}D,\\ \,\,{\omega }_{h}=\gamma {h}_{0},\\ \,\,{v}_{{\rm{s}}{\rm{w}}}^{2}={\gamma }^{2}{L}_{0}^{2}\,Jq,\\ \mathop{h}\limits^{ \sim }(z,t)=\frac{h(z,t)}{{h}_{0}}.\end{array}$$

The first term on the right-hand side in equation (8) represents the linear torque, and the second term on the right-hand side represents the newly derived nonlinear torque induced by the interaction of the spin deflection driven by the first pump pulse and the light-induced effective field of the second pulse. We see that this nonlinear torque is proportional to the Dzyaloshinskii–Moriya interaction *D*, the effective field and the spin deflection. Thus, such a nonlinear torque acts only when the external excitation couples with magnons in an out-of-equilibrium altermagnetic system.

As the laser pulse duration is much shorter than the spin precession period, we may approximate the pulses to act as a Dirac delta function^27,43^, arriving at time *t*_1_ and *t*_2_.

We assume that the effective fields of both pulses *h*_1_ and *h*_2_ are confined to the same distance *d* near the boundary:

10 $${h}_{1,2}(z,t)={h}_{0}{{\rm{e}}}^{-z/d}\delta (t-{t}_{1,2}).$$

We have denoted the spin deflections induced by pump 1 and 2 as *φ*_1_ and *φ*_2_, respectively. Note that in between the arrival of the first and second pump and changing the indices 2 to 1, we retrieve the linear Klein–Gordon equation, as *φ*(*t* < *t*_1_) = 0.

#### Detection of the spin dynamics

The spin dynamics is probed in a similar manner to the experiment of ref. ^26^, so here we expand on this detection formalism to explain the observed modulation frequencies.

First, we remind ourselves of the general expression of the magneto-optical polarization rotation of the reflected light induced by the magnetization near the surface^44,45^.

11 $${\theta }_{K}=i\frac{a{k}_{0}^{2}}{2k}\frac{{t}_{0}\widetilde{{t}_{0}}}{{r}_{0}}{\int }_{0}^{\infty }{\rm{d}}z{\prime} {e}^{2ik{z}^{{\prime} }}M(z,t).$$

When we neglect the nonlinear torque, we know that the spin deflections induced by the first pump pulse are described by^27^:

12 $${\varphi }_{1}(z,t)={\int }_{-\infty }^{\infty }[\,{f}_{1}(\omega ){{\rm{e}}}^{-i{k}_{{\rm{sw}}}(\omega )z}+{p}_{1}(\omega ){{\rm{e}}}^{-z/d}]{{\rm{e}}}^{i\omega t}{\rm{d}}\omega .$$

The first term in equation (12) corresponds to the freely propagating solution, and the second term refers to pump-driven uniform precession. Note that this spin-wave solution is obtained analytically in the Fourier domain, and the integral represents the inverse Fourier transformation to the time domain. From the analytical solution in the Fourier domain and by using the exchange boundary conditions^46^, we found the expressions for ${f}_{1}(\omega )$ and ${p}_{1}(\omega )$ for the case of the impulsive excitation:

13 $$\begin{array}{l}{p}_{1}(\omega )=\frac{-i\omega {\omega }_{h}\widetilde{h}(\omega )}{-{\omega }^{2}+{\omega }_{0}^{2}+2i\alpha \omega -{v}_{\mathrm{sw}}^{2}/{d}^{2}},\\ {f}_{1}(\omega )=\frac{1/d-\xi }{\xi -i{k}_{\mathrm{sw}}(\omega )}{p}_{1}(\omega ).\end{array}$$

To calculate the magneto-optically detected spin deflection, it is first necessary to obtain the solution to the nonlinear Klein–Gordon equation for the spin deflections (equation (8)). The solution to the linear equation is known, and will simply add another propagating wave starting at *t*_2_ through interference, yielding only a phase factor ${{\rm{e}}}^{i\omega {t}_{2}}$. Therefore, we focus on solving the equation for only the nonlinear torque, which can be found analytically by transforming the equation to the Fourier domain.

14 $$\left(-{\omega }^{2}+{\omega }_{0}^{2}+2i\alpha \omega -{v}_{\mathrm{sw}}^{2}{\nabla }^{2}\right){\varphi }_{2}\left(\omega ,z\right)={\omega }_{D}{\omega }_{h}{\varphi }_{1}\left(z,\tau \right){{\rm{e}}}^{-z/d}{{\rm{e}}}^{-i\omega \tau }.$$

We substitute the known linear solution for ${\varphi }_{1}(z,\tau )$ as the inverse Fourier transformation in equation (12) and the spatiotemporal profile of the effective field $h(z,t).$

The solution to equation (14) will have a similar form to the solution for the linear case, but the amplitudes are modified, and the effective field of this nonlinear torque will effectively be confined in a region *d*/2 from the material surface.

15 $${\varphi }_{2}\left(\omega \right)={f}_{2}\left(\omega \right){{\rm{e}}}^{-i{k}_{\mathrm{sw}}\left(\omega \right)z}+{p}_{2}\left(\omega \right){{\rm{e}}}^{-2z/d}.$$

The amplitude of the solution driven by the nonlinear torque ${p}_{2}(\omega )$ can be found and the amplitude of the freely propagating solution is obtained by defining a pinning parameter *ξ* that describes the restrictions of spin precession at the boundary, and applying the exchange boundary condition^27^.

Note that in the limit *ξ → ∞*, spin precession at the boundary is forbidden. After applying the exchange boundary conditions, we obtain the amplitudes for both components of the solution:

16 $$\begin{array}{c}{p}_{2}(\omega )=\frac{{\omega }_{D}{\omega }_{h}{\int }_{-\infty }^{+\infty }{p}_{1}(\varOmega ){{\rm{e}}}^{-i(\omega -\varOmega )\tau }{\rm{d}}\varOmega }{-{\omega }^{2}+{\omega }_{0}^{2}+2i\alpha \omega -4{v}_{{\rm{s}}{\rm{w}}}^{2}/{d}^{2}},\\ {f}_{2}(\omega )=\frac{2/d-\xi }{\xi -i{k}_{{\rm{s}}{\rm{w}}}(\omega )}{p}_{2}(\omega ).\end{array}$$

Note that the integral represents the inverse Fourier transformation. Finally, as our results are sensitive to the *m*_*x*_ mode in our experiment, we convert the derived deflection of the *l*_*y*_ component to the *m*_*x*_ component, using the expression:

17 $${m}_{x}(\omega )=\frac{1}{i\omega }\left({\omega }_{A}-\frac{{v}_{{\rm{sw}}}^{2}}{{\omega }_{E}}{\nabla }^{2}\right){l}_{y}(\omega ).$$

We remind ourselves that ${m}_{x}(z,t)\approx {M}_{0}\varphi (z,t).$ Now we substitute the solutions (15) and (16) into equation (11), to obtain the 2D spectrum of the magneto-optical detection experiment, as a function of *Ω* and *ω*:

18 $${\theta }_{K}^{p}(\omega ,\varOmega )=i\frac{a{k}_{0}^{2}}{2k}\frac{\mathop{{t}_{0}}\limits^{ \sim }{t}_{0}}{{r}_{0}}\frac{1}{i\omega }\left({\omega }_{A}-\frac{4{c}^{2}}{{d}^{2}{\omega }_{E}}\right)\left(\frac{1}{2k+2i/d}\right)\frac{{\omega }_{D}{\omega }_{h}{p}_{1}(\varOmega ){{\rm{e}}}^{-i\omega \tau }}{-{\omega }^{2}+{\omega }_{0}^{2}+2i\alpha \omega -4{v}_{{\rm{s}}{\rm{w}}}^{2}/{d}^{2}}$$

and

19 $$\begin{array}{c}{\theta }_{K}^{f}(\omega ,\varOmega )=i\frac{a{k}_{0}^{2}}{2k}\frac{\mathop{{t}_{0}}\limits^{ \sim }{t}_{0}}{{r}_{0}}\frac{1}{i\omega }\left({\omega }_{A}+\frac{{c}^{2}{k}_{{\rm{s}}{\rm{w}}}{(\omega )}^{2}}{{\omega }_{E}}\right)\left(\frac{2/d-\xi }{\xi -i{k}_{{\rm{s}}{\rm{w}}}(\omega )}\right)\\ \times \left(\frac{1}{2k-{k}_{{\rm{s}}{\rm{w}}}(\omega )}\right)\frac{{\omega }_{D}{\omega }_{h}{p}_{1}(\varOmega ){e}^{-i\omega \tau }}{-{\omega }^{2}+{\omega }_{0}^{2}+2i\alpha \omega -4{v}_{{\rm{s}}{\rm{w}}}^{2}/{d}^{2}}.\end{array}$$

This expression is plotted in Fig. 4, and shows the peaks at the diagonal frequencies as a result of interference and the peak at the off-diagonal as a result of the magnon conversion by the nonlinear torque. In our calculation, we used the parameters shown in Extended Data Table 1.

Note that the light-induced effective field used in our simulations is relatively small compared to that of previous reports^47^, owing to the low pump fluence used in our experiment. For simplicity, we have derived all of the above equations assuming the Brillouin conditions for normal incidence. Although the parameters of *n* and *β* are not explicitly specified in our equations, they are used for the proper projection of the probe wave vector on the magnon wave vector to use the Brillouin condition for oblique incidence:

20 $$2kn\,\cos (\beta )={k}_{{\rm{sw}}}.$$

### Altermagnetic symmetry of orthoferrites

Here we show that the orthoferrites satisfy the criteria to be classified as altermagnets. The criteria formulated in ref. ^10^ are as follows: “there is an even number of magnetic atoms in the unit cell”, “there is no inversion centre between the sites occupied by the magnetic atoms” and “the two opposite-spin sublattices are connected by crystallographic rotation transformation (may be combined with translation or inversion transformation)”.

The orthoferrites have four Fe atoms in the unit cell, thus satisfying the first criterion. The Fe ions occupy inversion centres and there is no inversion centre between them; hence, the second criterion is satisfied as well. Finally, the antiferromagnetic structure of HoFeO_3_ in the Γ_2_ phase, relevant for the present experiment, is invariant with respect to the screw axis transformation (that is, rotation around the *x* axis plus translation), satisfying the third condition. More details on the symmetry of the orthoferrites can be found in ref. ^48^. Moreover, altermagnets may be classified on the basis of their strong magneto-optical responses^13^. One of the characteristic features of the orthoferrites is their strong magneto-optical and optomagnetic responses^47^. For instance, the Faraday effect in the orthoferrites has been shown to scale linearly with the magnetic order parameter **L** (ref. ^49^), which is a manifestation of altermagnetism.

### HoFeO_3_ material properties and sample information

Holmium orthoferrite (HoFeO_3_) is a weak ferromagnet, with antiferromagnetically ordered spins below the Neel temperature of approximately 650 K (ref. ^50^). The non-vanishing Dzyaloshinskii–Moriya interaction slightly cants the otherwise antiparallel spins, thus resulting in a weak net ferromagnetic moment. The canting angle in the weak ferromagnets is proportional to the Dzyaloshinskii–Moriya interaction constant *D* (ref. ^51^). Similar to the other orthoferrites, HoFeO_3_ is an insulator, with a bandgap energy, *E*_g_, of about 3 eV (ref. ^52^). As a result, the orthoferrite exhibits a strong absorption of photons with an energy higher than this bandgap. The absorption enables the nanoscale confinement of the optical excitation of spins next to the sample facet and is essential for generating a propagating wave packet of magnons^26^.

HoFeO_3_ is a unique orthoferrite in the sense that its magnetic phase structure is complex, having more phases of spin orientation than the other orthoferrites. Here we summarize its magnetic properties. The magnetic structure is described by the antiferromagnetic vector **L** = **M**_1_ − **M**_2_ and the ferromagnetic vector **M** = **M**_1_ + **M**_2_. The magnetic phases are defined by the three temperatures *T*_1_ ≈ 38 K, *T*_2_ ≈ 52 K and *T*_3_ ≈ 58 K (ref. ^53^). At low temperatures *T* < *T*_1_, the Fe spins are in the Γ_2_ phase, in which the ferromagnetic moment **M** aligns along the crystallographic *a* axis, and the antiferromagnetic moment **L** aligns along the *c* axis. At high temperatures *T* > *T*_3_, the spin system enters the Γ_4_ phase, in which **M** aligns along the *c* axis and **L** aligns along the *a* axis. In between these temperatures, **L** gradually rotates from the *c* axis to the *a* axis, first through the *b*–*c* plane (Γ_12_ phase, *T*_1_ < *T* < *T*_2_) and then through the *a*–*c* plane (Γ_24_ phase, *T*_2_ < *T* < *T*_3_). HoFeO_3_ features extremely low damping of magnon modes^54^ and strong linear magneto-optics^49^, which is one of the signatures of altermagnetism^55^.

The HoFeO_3_ sample measured in our experiments is *c*-cut and has a thickness of about 60 μm. Our experiments are typically carried out at temperatures close to the temperature *T*_1_, such that the equilibrium weak ferromagnetic moment is aligned along the *a* axis. The sample’s *a* axis is oriented horizontally, along with a small magnetic field to saturate the domains.

### Experimental setup

A detailed layout of the experimental setup is depicted in Extended Data Fig. 1.

The HoFeO_3_ sample is placed in an open-cycle cryostat, which is cooled with liquid helium, down to temperatures of 5 K. The temperature is regulated by controlling the helium flow and the heat applied using a temperature controller. We use an electromagnet to apply a small magnetic field of 25 mT to saturate the magnetic domains. The laser pulses are generated by a Spectra-Physics Ti:sapphire laser amplifier, which outputs photons with a wavelength of 800 nm, with a repetition rate of 1 kHz. Most of the generated 800-nm light is guided into an optical parametric amplifier, which converts the 800-nm light into photons with other wavelengths. The optical parametric amplifier allows us to tune the photon energy in the UV–Vis–NIR range. The remainder of the 800-nm light is attenuated and guided through a delay line, and after attenuation with neutral density filters illuminates a BBO crystal that converts the 800-nm photons to 400-nm photons through second-harmonic generation. The residue of the 800-nm light is removed with a Schott BG39 filter. The intensity of the pump pulses can be tuned with a combination of polarizer and half-wave plate. The linear polarization of the light is rotated with a half-wave plate (*λ*/2). A lens is placed such that the sample is nearly in the focal plane of the lens. The 400-nm light excites the spin dynamics in the HoFeO_3_.

We probe the spin dynamics magneto-optically by measuring the polarization rotation of the probe light reflected from the sample. For our probe pulse, we use the output of the optical parametric amplifier. Typically, in our experiments we choose 660 nm for the probe wavelength. This light is tightly focused in the area illuminated by the pump pulse. We confirmed with a knife edge measurement that the focal spot of the probe pulse (≈100 μm) is much smaller than the focal spot of the pump pulse (≈1 mm). Typically, we pump with a pulse energy of approximately 0.8 μJ, which results in a fluence of approximately 0.1 mJ cm^−2^. The reflection from the surface of the sample is captured and collimated by a second lens. The light is guided and focused into a homemade pair of balanced photodetectors. A Wollaston prism is used to separate the orthogonal polarizations of the light.

The dynamical Kerr rotation is obtained by tracking the amplified difference of the signals in the photodiodes as a function of the time delays. The signals are analysed with a lock-in amplifier. The lock-in reference is coupled to the modulation frequency of the pump (500 Hz) and allows our results to be sensitive only to the pump-induced changes of the signal.

For our double-pump experiment, we use a beam splitter to split the pump pulse into two pump pulses, in a Michelson interferometer arm. The distance between the mirrors and beam splitters is adjustable, thereby allowing variation of the time delay between the two pumps. For convenience, we refer to the two orthogonal interferometer arms as stage 1 and stage 2. We carry out our double-pump experiment in two distinct configurations. In the first configuration, the chopper is placed after the interferometer arms (chopper 2). As a result, both pumps are modulated at 500 Hz, and our results are sensitive to the signal induced by both pumps.

In the other configuration, the chopper is placed in one of the arms of the Michelson interferometer (stage 1, chopper 1). In this configuration, only the pump pulses travelling through this arm are modulated at the 500-Hz lock-in reference frequency. Hence, in our measurements, we directly observe only the signal induced by the pump from stage 1. The pump from stage 2 affects only the signal from stage 1, but we cannot directly detect the pump-induced signal from this stage. Hence, our results are sensitive only to the nonlinear modulations of this second pump, and linear effects such as the coherent superposition of the spin waves launched by the separate pumps are not observable in this configuration.

**Extended Data Fig. 2:**
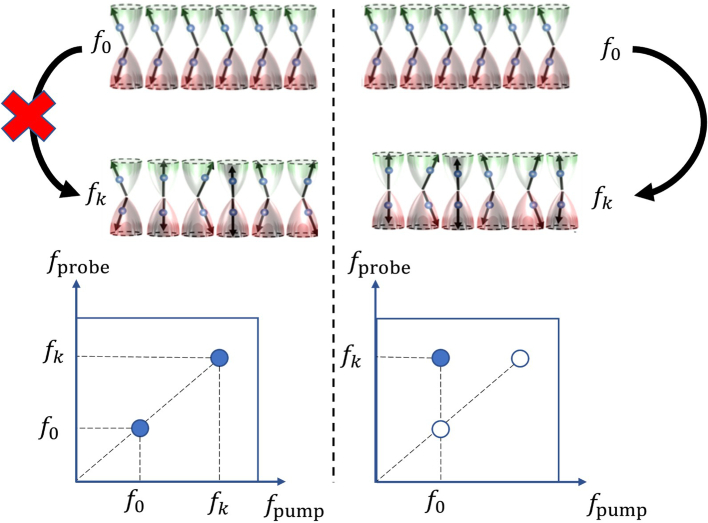
Concept of the 2D spectroscopy experiment. In the linear regime, the spin waves are modulated by interference only. As a result, upon performing the 2D Fourier Transform along the delay of the first pump pulse with the probe pulse (*f*_probe_) and the second pump pulse (*f*_pump_) diagonal features at (*f*_0_,* f*_0_) and (*f*_*k*_,* f*_*k*_) in the 2D spectrum are obtained. In contrast, if the amplitude of the mode oscillating at frequency *f*_*k*_ is modulated at frequency *f*_0_, the additional off-diagonal peak feature at (*f*_0_,* f*_*k*_) emerges.

**Extended Data Fig. 3:**
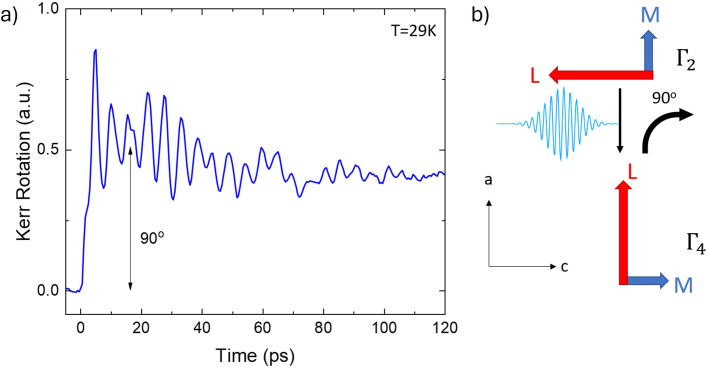
Calibration curve for the spin deflection with the help of the photo-induced spin reorientation transition from the Γ_2_ to the Γ_4_ phase. a) Time trace of the Kerr rotation measured for maximum pump fluence, such that the step due to spin reorientation transition was saturated. b) Illustration of the spin reorientation responsible for the step in panel a). This step can be attributed to the rotation of the magnetization from the x-axis (in plane) to the z-axis (out of plane). We approximate the deflection angle with the ratio of the oscillation amplitude and the step and find that this angle is approximately 15 degrees. We note that the direction of the spin reorientation is determined by the polarization of the pump pulse, as has been previously reported in ref. ^53^.

**Extended Data Fig. 4:**
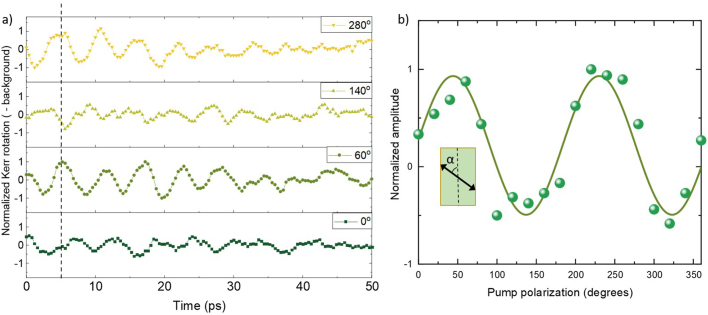
Investigation of the excitation mechanism from the pump polarization dependence. Panel a) shows the residual Kerr rotation for a variety of incident pump polarizations and panel b) shows the tracked amplitude of the oscillations in a) as function of the pump polarization. The oscillation amplitude depends strongly on the incident polarization, with the maximum amplitude occurring at diagonal polarization between the crystallographic a- and b axis. Therefore, the excitation mechanism most strongly matches to the Inverse Cotton-Mouton Effect symmetry. Thus, the spin waves are launched through non-thermal effects, in the form of Impulsive Stimulated Raman (or Brillouin) Scattering.

**Extended Data Fig. 5:**
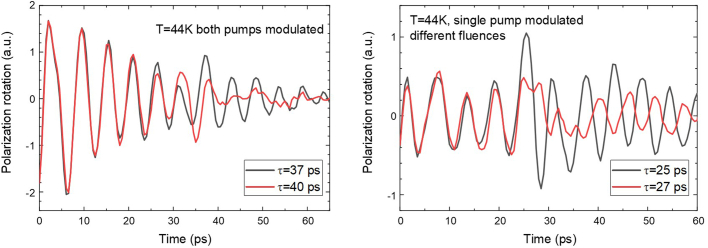
Comparison of the amplitudes of the spin waves before and after arrival of the second pump pulse. The left panel shows example data for the case when both pumps are modulated (main text Fig. 3), the right panel shows the data for the case of the single pump modulated with different fluences for both pumps (Extended Data Fig. 8d). We observe identical dynamics before the arrival of the 2nd pulse and the modulation only occurs after the arrival of the second pump pulse. We confirm the amplification or suppression of the spin wave, depending on the time delay between the two pump pulses. Note that beside the amplitude, the phase of the resulting spin wave can also be controlled by variation of the delay between the pump pulses.

**Extended Data Fig. 6:**
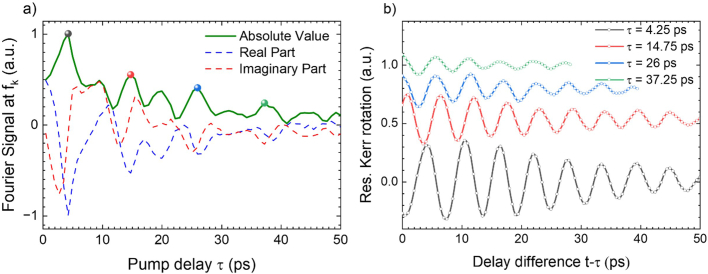
Estimation of the amplitude of the nonlinear conversion compared to the direct excitation amplitude. a) Magnitude, real and imaginary part of the Fourier transform with respect to the probe delay t, at the frequency f_k_. b) Comparison of the signals at various pump delays indicated in the left panel with the spheres. At long pump delays, we expect the spin precession to be damped, thus decreasing the contribution of the nonlinearity to the amplitudes of the signals. Indeed, we see that for the long time delays, the phase of the oscillations does not change, implying that interference is the main contributor to the oscillation amplitude. One can also observe that the amplitude of the conversion is in the same order of magnitude as the direct excitation of propagating magnons.

The concept of such a 2D spectroscopy experiment is illustrated in Extended Data Fig. 2. In the left-hand side of the figure, we consider the case of only interference, or no interaction between the modes. We see that the amplitude of the spin wave will be modulated only at the frequencies of the modes themselves, resulting in the emergence of the diagonal peaks (*f*_0_, *f*_0_) and (*f*_*k*_, *f*_*k*_). On the other hand, if a peak appears at the off-diagonal (*f*_0_, *f*_*k*_) as in our experiments, this indicates that the amplitude of the modes oscillating at frequency *f*_*k*_ is modulated with frequency *f*_0_. Such an effect can be explained only as being due to the presence of a nonlinear torque due to the coupling between the photon and the magnon. As compared to a single-pump experiment, the second pump pulse exerts an additional torque on the already deflected spin that allows the generation of oscillations at frequencies *f*_*k*_ from the frequency *f*_0_, thus up-conversion. We note that this up-conversion is impossible in the single-pump experiment, as the duration of the excitation is much shorter than the precession period.

### Analysis procedure

We measure 2D scans by first setting the pump delay stage to a fixed time delay, and measuring the spin dynamics by varying the probe delay stage. In our experimental setup (Extended Data Fig. 1), the light used as the probe has a fixed arrival time. The pumps pass through a delay line and are split in a Michelson interferometer, in which one of the arms is static and the other is moved to vary the time delay between the pumps. In this configuration, varying the time delay between the two pumps will also affect the temporal overlap between pump 2 and the probe. Hence, care should be taken when creating the 2D Fourier spectra.

As the measured signals are commonly associated with a step after the arrival of the second pump (due to light-induced phase transition), this offset distorts the Fourier analysis by adding zero-frequency components. We remove this offset by fitting data after the step with a polynomial, and subtract this fit from the data. We shift our starting point of the fit according to the delay between the two pumps, such that only the data after arrival of the second pump are fitted. In the main text, these subtracted data are shown in Fig. 3a. Although our focus is on the modulation after the arrival of the second pump, in the following section we also compare the amplitudes of the spin waves before and after the arrival of the second pump to illustrate the induced modulation by the second pump.

We checked for the occurrence of any artefacts in our analysis. For the case when both pumps are modulated, we checked this by taking the two separate reference scans obtained by measuring with the single pumps from both arms of the Michelson interferometer. We fit the data a few picoseconds after the pump–probe overlap, to remove low-frequency artefacts occurring due to the step. We temporally shift one of these reference scans according to the experimental pump delays while keeping the other scan static, and add both the scans for each experimentally used time delay between the pumps, thus creating a temporal 2D map. We carry out the 2D Fourier transform of these data, to obtain the 2D spectrum with respect to pump and probe delay, as shown in Fig. 3d. From this analysis procedure we obtained a single diagonal peak corresponding to the interference of the two spin waves, indicating that the off-diagonal peaks we found are not a result of artefacts in our analysis procedure.

**Extended Data Fig. 7:**
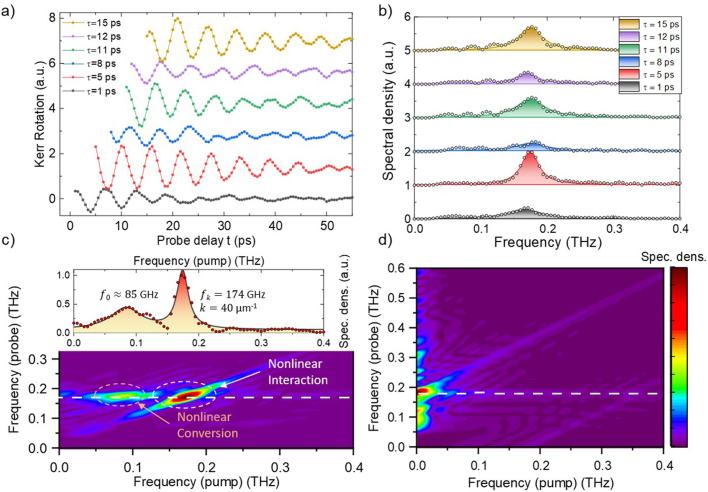
Experimental results of the double pump experiment with only a single pump modulated. a) Normalized probe polarization rotation traces, for various delays between the 2 pump pulses. Only one of the pumps is modulated and the data are shown after the arrival of the second pump pulse. The non-oscillatory background signal is subtracted. b) Fourier transforms of the traces in a). c) 2D Fourier transform of the data measured in a), with the Fourier transform along the probe delay on the y-axis and the Fourier transform along the pump delay on the x-axis. The upper part shows the spectrum along the pump frequency at the magnon frequency detected by the probe. d) Reference 2D Fourier transform obtained by adding two single pump reference scans. The data were measured with similar conditions as in Fig. 3 of the main text, but with only a single pump beam modulated by the optical chopper. Therefore, this experimental configuration removes sensitivity to the effect of linear interference. The observation of the off-diagonal feature in this experiment confirms its nonlinear nature. Moreover, nonlinear modulation of the *f*_*k*_ magnon mode is also present and may originate from a similar nonlinear effect as the *f*_0_ to *f*_*k*_ magnon up-conversion.

**Extended Data Fig. 8:**
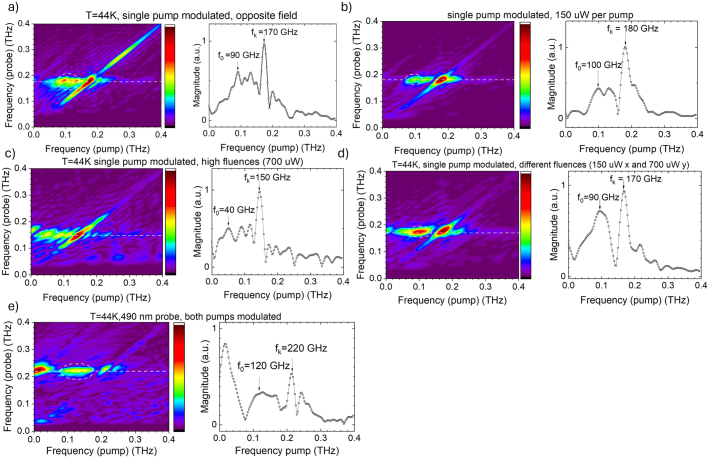
2D spectral maps for various experimental conditions. a) Similar conditions as the data in the main text, but with opposite polarity of the external magnetic field. b) T = 44 K, one modulated pump, and low fluences of 0.04 mJ/cm^2^ of both pumps. c) T = 44 K, one modulated pump, and high fluences of 0.2 mJ/cm^2^ of both pumps. d) T = 44 K, one modulated pump, and mixed fluences of 0.04 mJ/cm^2^ for the modulated pump, and 0.2 mJ/cm^2^ for the other pump. e) T = 44 K, both pumps modulated, and a probe wavelength of 490 nm. In these data sets, we see a consistent appearance of the off-diagonal peak at the *f*_0_ quasi-uniform spin precession frequency. We observed that this non-linearity is most pronounced at temperatures close to the spin reorientation transition temperature, for which *f*_k_ ≈ 2*f*_0_. We may attribute small frequency shifts when comparing data for different fluences due to the effect of cumulative heating by the train of the laser pulses.

For the case when the single static pump is modulated, we again created a temporal 2D map. As the signals from the time-shifted pump are not visible in this configuration, we have the signal for each time delay. We carry out the 2D Fourier transform and obtain the spectrum in Extended Data Fig. 7d. As expected, we observe no modulation in this reference scan, resulting in only zero-frequency features along the pump delay. The absence of any diagonal and off-diagonal peaks indicates that our off-diagonal peak is explained by a physical nonlinearity. It also highlights that the observed diagonal peak in Extended Data Fig. 7c may be explained by a similar nonlinearity.

## Online content

Any methods, additional references, Nature Portfolio reporting summaries, source data, extended data, supplementary information, acknowledgements, peer review information; details of author contributions and competing interests; and statements of data and code availability are available at 10.1038/s41586-024-07448-3.

### Source data

Source Data Fig. 2

Source Data Fig. 3

Source Data Fig. 4

## Figures and Tables

**Extended Data Table 1:**
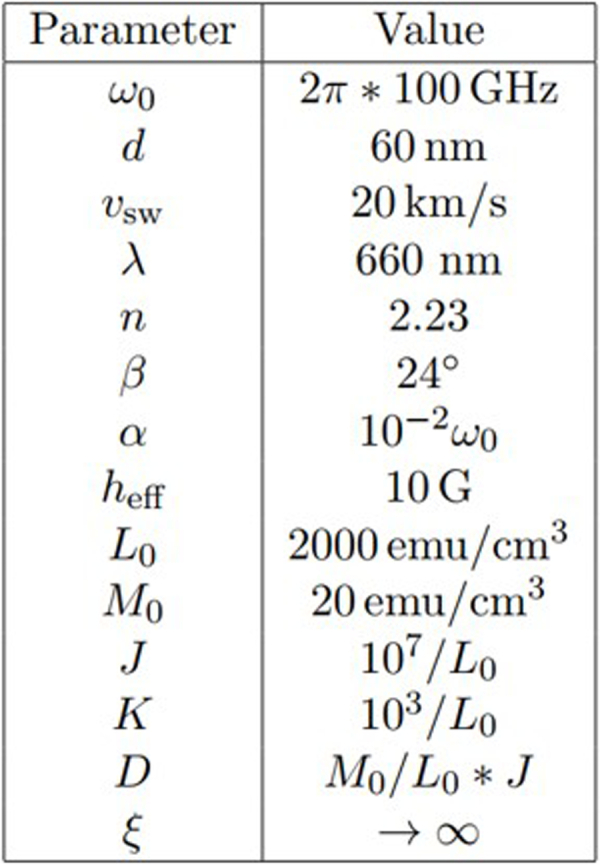
Overview of parameters used in the simulation of the 2-dimensional detected dynamics spectrum

## Data Availability

Raw data and source data for the figures are publicly available at 10.5281/zenodo.10895423 (ref. ^56^). Source data are provided with this paper.

## Code availability

Codes used for the data analysis and the numerical simulations are publicly available at 10.5281/zenodo.10895423 (ref. ^56^).

## References

[1] Jungwirth T, Marti X, Wadley P, Wunderlich J (2016). Antiferromagnetic spintronics. Nat. Nanotechnol..

[2] Nemec P, Fiebig M, Kampfrath T, Kimel AV (2018). Antiferromagnetic opto-spintronics. Nat. Phys..

[3] Lebrun R (2020). Long-distance spin-transport across the Morin phase transition up to room temperature in ultra-low damping single crystals of the antiferromagnet α-Fe2O_3_. Nat. Commun..

[4] Lebrun R (2018). Tunable long-distance spin transport in a crystalline antiferromagnetic iron oxide. Nature.

[5] El Kanj A (2023). Antiferromagnetic magnon spintronic based on nonreciprocal and nondegenerated ultra-fast spin-waves in the canted antiferromagnet α-Fe_2_O_3_. Sci. Adv..

[6] Krempaský J (2024). Altermagnetic lifting of Kramers spin degeneracy. Nature.

[7] Lee S (2024). Broken Kramers degeneracy in altermagnetic MnTe. Phys. Rev. Lett..

[8] Zhu Y-P (2024). Observation of plaid-like spin splitting in a noncoplanar antiferromagnet. Nature.

[9] Reimers S (2024). Direct observation of altermagnetic band splitting in CrSb thin films. Nat. Commun..

[10] Šmejkal L, Sinova J, Jungwirth T (2022). Emerging research landscape of altermagnetism. Phys. Rev..

[11] Cheong S-W, Huang F-T (2024). Altermagnetism with non-collinear spins. npj Quant. Mater..

[12] Kimel, A., Rasing, T. & Ivanov, B. Optical read-out and control of antiferromagnetic Neel vector in altermagnets and beyond. Preprint at https://arxiv.org/abs/2403.10129 (2024).

[13] Mazin I, The PRX Editors (2022). Editorial: altermagnetism—a new punch line of fundamental magnetism. Phys. Rev..

[14] Chumak AV, Vasyuchka VI, Serga AA, Hillebrands B (2015). Magnon spintronics. Nat. Phys..

[15] Barman A (2021). The 2021 magnonics roadmap. J. Phys. Condens. Matter.

[16] Kolosvetov A, Kozhaev M, Savochkin I, Belotelov V, Chernov A (2022). Concept of the optomagnonic logic operation. Phys. Rev. Appl..

[17] Chumak AV, Serga AA, Hillebrands B (2014). Magnon transistor for all-magnon data processing. Nat. Commun..

[18] Koerner C (2022). Frequency multiplication by collective nanoscale spin-wave dynamics. Science.

[19] Baltz V (2018). Antiferromagnetic spintronics. Rev. Mod. Phys..

[20] Wadley P (2016). Electrical switching of an antiferromagnet. Science.

[21] Li JX (2020). Spin current from sub-terahertz-generated antiferromagnetic magnons. Nature.

[22] Vaidya P (2020). Subterahertz spin pumping from an insulating antiferromagnet. Science.

[23] Dąbrowski M (2020). Coherent transfer of spin angular momentum by evanescent spin waves within antiferromagnetic NiO. Phys. Rev. Lett..

[24] Das S (2022). Anisotropic long-range spin transport in canted antiferromagnetic orthoferrite YFeO_3_. Nat. Commun..

[25] Wang H (2023). Long-distance coherent propagation of high-velocity antiferromagnetic spin waves. Phys. Rev. Lett..

[26] Hortensius JR (2021). Coherent spin-wave transport in an antiferromagnet. Nat. Phys..

[27] Leenders RA, Mikhaylovskiy RV (2023). Theory of optical generation and detection of propagating magnons in an antiferromagnet. Phys. Rev. B.

[28] Nguyen TA, Nguyen TLT, Bui VX (2021). Influence of the synthetic conditions on the crystal structure, magnetic and optical properties of holmium orthoferrite nanoparticles. J. Mater. Sci. Mater. Electron..

[29] Bar’yakhtar VG, Ivanov BA, Chetkin MV (1985). Dynamics of domain walls in weak ferromagnets. Sov. Phys. Usp..

[30] Zvezdin AK (1979). Dynamics of domain-walls in weak ferromagnets. JETP Lett..

[31] Kalashnikova AM (2007). Impulsive generation of coherent magnons by linearly polarized light in the easy-plane antiferromagnet FeBO_3_. Phys. Rev. Lett..

[32] Šmejkal, L. et al. Chiral magnons in altermagnetic RuO_2_. *Phys. Rev. Lett.* **131**, 256703 (2023). 10.1103/PhysRevLett.131.256703 38181333

[33] Peceli D (2012). Optimization of the double pump-probe technique: decoupling the triplet yield and cross section. J. Phys. Chem. A.

[34] Kimel AV, Kirilyuk A, Hansteen F, Pisarev RV, Rasing T (2007). Nonthermal optical control of magnetism and ultrafast laser-induced spin dynamics in solids. J. Phys. Condens. Matter.

[35] Zhang Z (2023). Generation of third-harmonic spin oscillation from strong spin precession induced by terahertz magnetic near fields. Nat. Commun..

[36] Zhang, Z. et al. Terahertz-field-driven magnon upconversion in an antiferromagnet.* Nat. Phys.*10.1038/s41567-023-02350-7 (2024).

[37] Li XW (2018). Observation of Dicke cooperativity in magnetic interactions. Science.

[38] Makihara T (2021). Ultrastrong magnon-magnon coupling dominated by antiresonant interactions. Nat. Commun..

[39] Mashkovich EA (2021). Terahertz light-driven coupling of antiferromagnetic spins to lattice. Science.

[40] Lu J (2017). Coherent two-dimensional terahertz magnetic resonance spectroscopy of collective spin waves. Phys. Rev. Lett..

[41] Blank TGH (2023). Empowering control of antiferromagnets by THz-induced spin coherence. Phys. Rev. Lett..

[42] Zvezdin, A. Dynamics of domain walls in weak ferromagnets. *JETP Lett.* **29**, 553 (1979).

[43] Tzschaschel C, Satoh T, Fiebig M (2020). Efficient spin excitation via ultrafast damping-like torques in antiferromagnets. Nat. Commun..

[44] Thomsen C, Grahn HT, Maris HJ, Tauc J (1986). Surface generation and detection of phonons by picosecond light pulses. Phys. Rev. B.

[45] Zvezdin, A. K. & Kotov, V. A. *Modern Magnetooptics and Magnetooptical Materials* (CRC Press, 1997).

[46] Gurevich, A. G. & Melkov, G. A. *Magnetization Oscillations and Waves* (CRC press, 1996).

[47] Kimel AV (2005). Ultrafast non-thermal control of magnetization by instantaneous photomagnetic pulses. Nature.

[48] Yamaguchi T, Tsushima K (1973). Magnetic symmetry of rare-earth orthochromites and orthoferrites. Phys. Rev. B.

[49] Afanasiev D (2017). Femtosecond single-shot imaging and control of a laser-induced first-order phase transition in HoFeO_3_. J. Phys. Condens. Matter.

[50] Stewart GA, Iles GN, Mole RA, Yamani Z (2023). An inelastic neutron scattering investigation of holmium orthoferrite. J. Phys. Condens. Matter.

[51] Mikhaylovskiy RV (2015). Ultrafast optical modification of exchange interactions in iron oxides. Nat. Commun..

[52] Kahn FJ, Pershan PS, Remeika JP (1969). Ultraviolet magneto-optical properties of single-crystal orthoferrites, garnets, and other ferric oxide compounds. Phys. Rev..

[53] Kimel AV (2009). Inertia-driven spin switching in antiferromagnets. Nat. Phys..

[54] Blank, T. G. H., Grishunin, K. A. & Kimel, A. V. Magneto-optical detection of terahertz cavity magnon-polaritons in antiferromagnetic HoFeO_3_. *Appl. Phys. Lett.* **122**, 072402 (2023).

[55] Kimel AV, Zvezdin AK (2022). Universal orthoferrites and orthoferrites as a universe. Photonics Insights.

[56] Leenders, R., Afanasiev, D., Kimel, A. & Mikhaylovskiy, R. Source data and codes for canted spin order as a platform for ultrafast conversion of magnons. *Zenodo*10.5281/zenodo.10895423 (2024). 10.1038/s41586-024-07448-3 PMC11168928 38811734

